# Vitamin D in liver cancer: novel insights and future perspectives

**DOI:** 10.3325/cmj.2022.63.187

**Published:** 2022-04

**Authors:** Antonio Markotić, Tomislav Kelava, Helena Markotić, Hrvoje Silovski, Anna Mrzljak

**Affiliations:** 1 Department of Physiology, University of Mostar School of Medicine, Mostar, Bosnia and Herzegovina; 2 Department of Physiology, University of Zagreb School of Medicine, Zagreb, Croatia; 3 Department of Internal Medicine, University Hospital Mostar, Mostar, Bosnia and Herzegovina; 4 Department of Surgery, University Hospital Center Zagreb, Zagreb, Croatia; 5 Department of Gastroenterology and Hepatology, University Hospital Center Zagreb, Zagreb, Croatia

## Abstract

Vitamin D has been a focus of attention in liver cancer due to its direct and indirect antineoplastic effects. This review critically evaluates data from recently published basic and clinical studies investigating the role of vitamin D in liver cancer. Basic studies indicate that vitamin D plays an important role in liver cancer development by suppressing the activity of hepatic stellate cells and Kupffer cells. Furthermore, vitamin D has a direct anti-proliferative, anti-angiogenic, proapoptotic, and prodifferentiative effect on liver cancer cells. Recent investigation suggested several interesting mechanisms of these actions, such as inactivation of Notch signaling, p27 accumulation, and tyrosine-protein kinase Met/extracellular signal-regulated kinases inhibition. On the other hand, data from clinical observational studies, although promising, are still inconclusive. Unfortunately, studies on the effect of vitamin D supplementation were generally focused on short-term outcomes of chronic liver diseases (liver enzyme levels or elastographic finding); therefore, there are still no reliable data on the effect of vitamin D supplementation on liver cancer occurrence or survival.

Numerous observational studies emphasized a possible link between vitamin D deficiency and cancer risk. The association was first established for cancers with a greater incidence in high-latitude regions, where vitamin D deficiency is more prevalent, such as colon cancer. Although liver cancers, due to a strong association with known risk factors such as hepatitis B (HBV) and C virus (HCV) and alcohol abuse, do not follow this latitude-related pattern, recent studies have suggested that vitamin D may still have a role in liver cancer development. This review critically evaluates data from basic and clinical studies investigating the possible role of vitamin D in liver cancer.

## Pathophysiological background

Principal liver cells, hepatocytes, usually express a very low level of vitamin D receptor (VDR) or none at all. However, VDR is highly expressed in nonparenchymal liver cells such as Kupffer cells, hepatic stellate cells, and sinusoidal endothelial cells, which play an important role in liver tumor development (1). The main effects of vitamin D on various liver cells are summarized in Table 1.

**Table 1:** The main effects of vitamin D or its analogs on various liver cells*

Cell type	Main effects
Hepatocytes	A very low level of VDR expression Expression is induced in NAFLD but decreased in NASH or chronic hepatitis C (8,9) VDR activation might be associated with lipid accumulation and contribute to steatosis development (8,10)
Kupffer cells	Abundant expression of VDR that exhibits anti-inflammatory effects upon activation: VDR activation suppresses the LPS-induced inflammation and downregulates IL-6, TNF, and IL-1b expression (2) VDR activation mitigates inflammatory response in macrophages following ER stress challenge (3)
Hepatic stellate cells	Significant VDR expression Vitamin D and its analogs exert inhibitory effects on primary murine hepatic cells or human cell lines, possibly through inactivation of TGF-β/Smad signaling (5-7)
Cholangiocytes	High VDR expression with immunoregulatory functions Ursodeoxycholic acid and vitamin D induce the expression of antimicrobial peptide cathelicidin through a VDR-dependent mechanism (12) VDR deficiency promotes cholestatic liver injury through disruption of biliary epithelial cell junctions (13) Vitamin D or its analog ameliorate liver injury through a VDR-independent pathway (16)
Liver cancer cells	VDR is expressed in human liver cancer cell lines and specimens of human HCC (1,22,23) KLF4 might play a pivotal role in the regulation of VDR expression in HCC (39) Supplementation with vitamin D or its analogs inhibits the proliferation of cancer cell lines and induces apoptosis through several mechanisms: disruption of HGF/c-met/ERK pathway due to downregulation of c-met and ERK (23) increase in E-cadherin and decrease in Akt expression (28) induction of cell cycle arrest through p27 accumulation (25) decreased HDAC2 with increased p21 (WAF1/Cip1) expression and subsequent modulation of p53, Bax, DR5, caspase 8, and Bcl-2 protein expressions (26,27) modulation of TLR7 expression and β-catenin activation (32) stimulation of TXNIP expression, inactivation of Notch signaling and/or p27(kip1)-dependent suppression of proinflammatory cytokines secretion (34-36)
Cholangiocarcinoma	VDR expression in human cholangiocarcinoma tissue specimens (41-43) Treatment with vitamin D or analogs impairs proliferation and induces apoptosis in cultured cells. Proposed mechanisms include induction of cell cycle arrest through regulation of cyclin D1, cyclin D3, CDK4, CDK6, p21, and/or p27 (44-47) VDR dependent downregulation of LCN2 expression (46,47,49)

*Abbreviations: CDK – cyclin dependent kinase; c-met – tyrosine-protein kinase Met; DR – death receptor; ER – endoplasmic reticulum; ERK – extracellular signal-regulated kinases; HCC –hepatocellular carcinoma; HDAC2 – histone deacetylase 2; HGF – hepatocyte growth factor; IL-6 – interleukin-6; KLF4 – Krüppel-like factor 4; LCN2 – lipocalin 2; LPS – lipopolysaccharide; NAFLD – nonalcoholic fatty liver disease; NASH – nonalcoholic steatohepatitis; Smad – mothers against decapentaplegic homologue; TGF-β – transforming growth factor beta; TLR – toll-like receptor; TNF – tumor necrosis factor; TXNIP – thioredoxin interacting protein; VDR – vitamin D receptor.

The most abundant expression of VDR in the liver was found in Kupffer cells. The effect of VDR activation in these cells is anti-inflammatory. The activation of VDR in Kupffer cells reduces the degree of lipopolysaccharide-induced activation by decreasing the secretion of interleukin (IL)-6, IL-1, and tumor necrosis factor alpha (TNF-alpha) (2). Similarly, VDR diminishes the induction of endoplasmatic reticulum stress by tunicamycin and subsequent inflammatory response (3). As pro-inflammatory milieu in the liver is associated with cancerogenesis, these mechanisms might explain one aspect of vitamin D anticancer properties.

Hepatic stellate cells, critical contributors to liver fibrosis, also express a significant amount of VDR. Various *in vitro* studies reported that inhibitory effect of VDR agonists on primary murine hepatic stellate cells (4) or human cell lines HSC-T6 and LX-2 (5-7) is mediated by a decrease in transforming growth factor-beta (TGF-β)/Smad signaling (7). These findings suggest that vitamin D may suppress liver fibrosis occurrence and progression, thereby decreasing liver cancer risk.

Although healthy hepatocytes do not express a significant amount of VDR, the expression may change in certain diseases. Hepatocyte expression of VDR was induced in non-alcoholic fatty liver disease (NAFLD) (8) and decreased in non-alcoholic steatohepatitis (NASH) or chronic hepatitis C (8,9). However, VDR activation in hepatocytes could promote lipid accumulation and contribute to steatosis development (8-10). Whether VDR effects on nonparenchymal cells override the potentially harmful effects of VDR stimulation in parenchymal cells is still controversial.

Cholangiocyte expression of VDR is high (1,11). VDR activation regulates the expression of the antimicrobial peptide cathelicidin in biliary epithelial cells, and ursodeoxycholic acid and vitamin D induce cathelicidin expression through a VDR-dependent mechanism (12). The absence of VDR aggravates cholestatic liver injury in mice through disruption of biliary epithelial cell junctions (13). VDR deficiency might also promote sustained inflammatory response in primary biliary cholangitis (14). Furthermore, the vitamin D/VDR pathway affected the extent of injury and fibrosis in a mouse model of sclerosing cholangitis (Abcb4 knockout mice) (15,16). Mice on a low-vitamin D diet exhibited a higher level of fibrosis (15). In contrast, VDR knockout mice had an increased cholestatic liver injury level and a significant lifespan reduction (16).

## Liver cancer models and liver cancer cell lines

During the last decades, several vitamin D properties that may hamper cancer development and growth have emerged, such as anti-proliferative, anti-inflammatory, anti-angiogenic, proapoptotic, and prodifferentiative effect (17-19). However, in terms of liver cancer cells, the most prominent effect of vitamin D is the inhibition of proliferation.

Multiple *in vitro* and *in vivo* studies have shown that supplementation with either vitamin D or vitamin D analogs inhibits the proliferation of various liver cancer cell lines and reduces the size of the tumors in mice (20-28). The anti-proliferative effect could be ascribed to disruption of hepatocyte growth factor/tyrosine-protein kinase Met/extracellular signal-regulated kinases (HGF/c-met/ERK) signaling pathway by vitamin D-induced downregulation of c-met and ERK (23), modulation of E-cadherin, and Akt expression (28), and/or induction of cell cycle arrest presumably due to p27 protein accumulation (25). Furthermore, treatment with vitamin D decreases the expression of histone deacetylase 2 (HDAC2) and increases the expression of p21 (WAF1/Cip1) in HepG2 cells, resulting not only in decreased cell growth but also in the induction of apoptotic cell death (26). Apoptosis could be induced through extrinsic and intrinsic pathways, as vitamin D treatment upregulates death receptor 5 and Bax protein expressions along with Bcl-2 downregulation. These findings were further confirmed *in vivo* as vitamin D-treated mice exhibited suppressed growth of xenograft human hepatocellular carcinoma (HCC) with a large area of necrosis (27).

Vitamin D may also affect TGF-β signaling in the liver. TGF-β is a pleiotropic cytokine that exhibits opposite functions depending on the context: it acts as a tumor suppressor in normal hepatocytes and early stages of tumorigenesis, but it can also promote tumor development in later stages, and it is highly expressed in HCC tissue (29-31). Vitamin D deficiency increases the tumor burden in TGF-β/Smad3-deficient mice through modulation of toll-like receptor 7 expression and β-catenin activation. Additionally, vitamin D supplementation restored the Smad3 expression in cirrhosis and HCC patients and reduced β-catenin expression in liver tissue of HCC patients, providing a rationale for vitamin D treatment in specific patients with disrupted TGF-β signaling (32). On the other hand, in later stages of the disease, the downregulation of TGF-β signaling may be beneficial. In this context, the finding that vitamin D treatment significantly reduces TGF-β level and Smad3, Snail, and matrix metalloproteinase-2 gene expression in experimental HCC model in rats, along with improvement of a histopathological picture, sounds promising (33).

Several other mechanisms involving vitamin D may attenuate carcinogenesis in the liver. Vitamin D stimulates the expression of thioredoxin-interacting protein (34) and inactivates Notch signaling in liver cancer cell lines, leading to anti-proliferative, anti-invasive, and proapoptotic effects (35). Vitamin D also decreases the secretion of pro-inflammatory cytokines from immune cells in a p27(kip1)-dependent way, hence undermining HCC development (36). The anti-tumor effect also may be exerted through the protection of hepatic progenitor cells. Vitamin D suppresses the aflatoxin B1-induced proliferation and dedifferentiation of liver progenitor cells (37).

VDR is expressed in both human liver cancer cell lines, especially HepG2 cell lines and specimens of human HCC (1,22,23). However, in certain circumstances, VDR expression may be reduced in liver cancer tissue, providing an escape mechanism from vitamin D effects (38,39). Furthermore, the tumor suppressor Krüppel-like factor 4 (KLF4) plays a pivotal role in regulating VDR expression in HCC. While decreased or lost KLF4 expression correlates with decreased VDR expression, overexpression of KLF4 upregulates VDR and sensitizes the cells to the vitamin D effects (39). Therefore, the cancer cell response to vitamin D treatment also depends on the expression of VDR in these cells. The finding that VDR expression might be upregulated by antihistamines, such as astemizole, which leads to the synergistic effect of astemizole and vitamin D (40), provides novel insights and confers the conclusion that additional basic studies are still warranted in order to elucidate detailed mechanisms and provide new targets for HCC treatment.

VDR expression was also found in human cholangiocarcinoma (CCA) tissue specimens (41-43). As in HCC, the treatment with vitamin D and/or vitamin D analogs showed beneficial effects in CCA cell cultures and *in vivo* models of CCA (41-47). Vitamin D or its analogs significantly impaired proliferation (41,42) and induced apoptosis in cultured cells (44), suppressed cholangiocarcinogenesis (43,48) and significantly inhibited tumor growth and progression in murine models of CCA (43,44,47). Several studies examined the mechanisms behind these effects, suggesting that vitamin D induces cell cycle arrest through regulation of cyclin D1 (44,45), cyclin D3 (47), p21 (44), and p27 expression (47). Furthermore, vitamin D/VDR signaling is involved in regulating the expression of lipocalin 2 (LCN2), an oncogene highly expressed in human intrahepatic cholangiocarcinoma tissue. Vitamin D significantly downregulates the expression of LCN2, which attenuates proliferation. This was further confirmed in LCN2 knockdown settings, where the loss of LCN2 made the cells less responsive to vitamin D or its analog treatment (46,47,49).

## Data from observational clinical studies

The relationship between vitamin D and predisposing diseases for liver cancer such as NAFLD, alcoholic liver diseases, or viral hepatitis has been extensively investigated, often with conflicting results (50,51). On the other hand, mounting data suggest that vitamin D deficiency reflects hepatic dysfunction, and as such, is associated with mortality in patients with liver cirrhosis, regardless of the underlying causes (52,53). In the context of liver cancer, higher VDR gene promoter methylation was detected in the HCC tissue (54). Data from vitamin D studies investigating liver cancer occurrence are summarized in Table 2.

**Table 2:** Studies investigating vitamin D and liver cancer occurrence

Incidence study	Number of patients	Key findings
Chinese Linxian Nutrition Intervention Trials (55)	255	modest evidence for associations with incident liver cancer, which became significant only among participants with higher baseline serum calcium
Nested case-control study within the European Prospective Investigation into Cancer and Nutrition (EPIC) cohort (57)	138	higher vitamin D levels were associated with a 49% reduction of HCC; the finding did not vary by time from enrolment to diagnosis, or changed after adjustment for biomarkers of preexisting liver damage or chronic HBV or HCV infection
Japan Public Health Center-based Prospective Study cohort (56)	110	vitamin D concentration was inversely associated with liver cancer, with corresponding hazard ratios for trend of 0.45 (0.26 to 0.79) (*P* = 0.006)
Sir Run Shaw Hospital, China (59)	100	vitamin D level greater than 20 ng/mL increased HCC risk (odds ratio 7.56, 95% confidence interval 4.58–12.50)

*Abbreviations: HCC – hepatocellular carcinoma; HBV – hepatitis B virus; HCV – hepatitis C virus.

The Chinese Linxian Nutrition Intervention Trials showed no significant associations between the risk of liver cancer occurrence and serum vitamin D levels. However, the risk estimates decreased across increasing quartiles of vitamin D concentrations (55). A large case-control study by Budhathoki et al (56) reported an inverse association between the pre-diagnostic vitamin D levels and liver cancer, which was independent of dietary factors or viral hepatitis infection. Interestingly, they found no association between vitamin D and other investigated cancers (gastric, rectal, colon, or lung cancer). Fedirko et al (57) previously reported similar findings in a nested case-control study within the EPIC cohort study. However, the EPIC cohort also demonstrated that a dairy-source vitamin D increased the risk of HCC. In contrast, a non-dairy source showed no association (58). Contrary to this, a recent study by Liu et al reported a higher vitamin D level among post-diagnostically sampled HCC patients (59). Except for the sampling time point, the studies further differed in many other important patient characteristics (diet, lifestyle, environmental exposures, and HCC risk factor profiles), which explains divergent results.

The prognostic value of vitamin D in liver cancer has been investigated in several trials (Table 3). A prospective German study showed that newly diagnosed HCC patients with serum total 25(OH)D≤10 ng/mL had significantly decreased overall survival compared with patients with 25(OH)D>10 ng/mL (60). A Finnish Alpha-Tocopherol, Beta-Carotene Cancer Prevention Study demonstrated that liver cancer patients with higher total 25(OH)D levels (up to 28 years before cancer diagnosis) had a suggestive, although not significant, improvement in liver cancer-specific survival (61). In a recent Chinese study, Fang et al (62) showed that higher serum bioavailable vitamin D levels (calculated from measured free vitamin D, albumin, and affinity constant between 25(OH)D and albumin) rather than total vitamin D levels were independently associated with improved survival. Data on vitamin D role in CCA are scarce. We identified two studies that reported better survival of patients with higher VDR expression in resected tumor tissue (42,47).

**Table 3:** Studies investigating vitamin D levels and liver cancer survival*

Survival study	Number of patients	Key findings
German Prospective cohort study (60)	200	low levels of vitamin D were associated with increased mortality risk from HCC independently of the MELD score and high AFP levels
Nested study form Alpha-Tocopherol, Beta-Carotene Cancer Prevention Study in Finnish smoker population (61)	206	higher levels of vitamin D were not significantly associated with better survival of liver cancer patients in a population of Finnish smokers
Guangdong Liver Cancer Cohort study (62)	1031	higher bioavailable vitamin D levels were significantly associated with better survival, independent of Barcelona Clinic Liver Cancer stage, cancer treatment, and serum C-reactive protein neither total nor free vitamin D levels were significantly associated with survival
*Abbreviations: AFP – alpha-fetoprotein; HCC – hepatocellular carcinoma; MELD – Model for End-Stage Liver Disease.		

The results of the studies on single nucleotide polymorphism further support the link between vitamin D and liver tumor development (Table 4). VDR polymorphism was associated with a risk of HCC occurrence in an alcoholic- (63), HCV- (64,65), and HBV- (66,67) related cirrhosis. There are still no published studies regarding the association between the VDR polymorphism and CCA.

**Table 4:** Studies on vitamin D-related single nucleotide polymorphism and liver tumor development

Reference	Etiology/ population/ N	VDR SNPs	Key points
Falleti et al (63)	HCV, HBV, ALD/ Italian/80 HCC, 236 healthy controls	VDR gene FokI BsmI ApaI TaqI	Association with HCC was found for b/b genotype of BsmI, T/T genotype of *Taq*I, absence of the A-T-C protective allele of BAT, and carriage of the BAT A-T-C and G-T-T haplotypes
Hoan et al (66)	HBV/Vietnamese/171 HCC, 183 CHB, 89 LC, 238 healthy controls	VDR gene FokI BsmI ApaI TaqI	ApaI CA genotype is less frequent, and APAL AA is more frequent in HCC vs CHB patients No association between TaqI, FokI, and BsmI polymorphisms and any clinical outcome was found
Barooah et al (65)	HCV/ Indian/ 60 HCC, 167 CHC, 124 LC, 102 healthy controls	VDR BsmI ApaI TaqI	ApaI CC genotype, ApaI C allele, and bAt haplotype were significantly associated with liver cancer paI CC genotype and bAt haplotype were independent predictors of HCC development
Rafat Rowida et al (64)	HCV/ Egyptian/ 80 HCC, 80 LC, 80 healthy controls	VDR gene Apa1	Apa1 CC is associated with greater risk for HCC development. It is also associated with a more severe Child-Pugh score and MELD score (*P* < 0.05)
Peng et al (67)	HBV/ Chinese/ 184 HCC, 296 HBV non-HCC, 180 healthy controls	VDR gene Fok1 rs3782905 Cdx2 DBP gene rs7041	Fok1 T allele and rs7041 G allele were associated with a significantly increased HBV-related HCC risk no significant effect of VDR rs11568820, and rs3782905 polymorphisms on HBV-related HCC risk

*Abbreviations: ALD – alcoholic liver disease; CHB – chronic hepatitis B, CHC – chronic hepatitis C; HCC – hepatocellular carcinoma; HBV – hepatitis B virus; HCV – hepatitis C virus; LC– liver cirrhosis; MELD – Model for End-Stage Liver Disease; SNP – single nucleotide polymorphism; VBP– vitamin D binding protein; VDR – vitamin D receptor.

## Data from clinical supplementation studies

In the last five years, numerous studies have investigated the effect of vitamin D supplementation on various chronic liver diseases. However, these studies were generally focused on short-term outcomes (liver enzyme levels or elastographic finding), and we found no reliable data on the effect of vitamin D supplementation on liver cancer occurrence or survival. The results are limited to one uncontrolled trial that suggested a weak effect of vitamin D analog, seocalcitol, in patients with inoperable HCC, and a pilot study that reported serious adverse effects in CCA patients treated with high-dose calcitriol in combination with chemotherapeutic drugs (68,69).

## Conclusion

**Figure 1:**
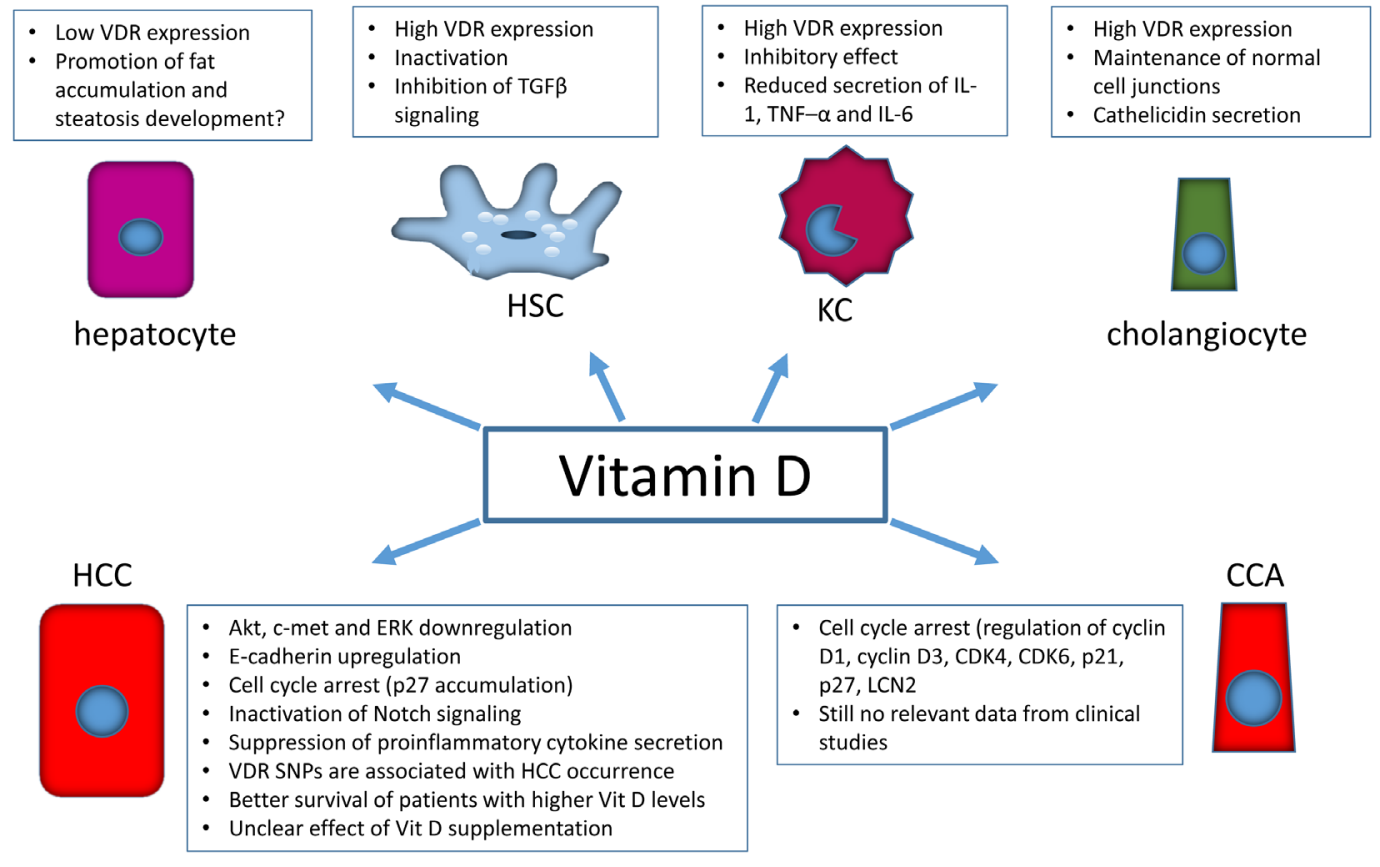
Summary of main effects of vitamin D on processes involved in liver cancer development. CCA – cholangiocarcinoma; CDK – cyclin dependent kinase; c-met – tyrosine-protein kinase Met; ERK – extracellular signal-regulated kinases; HCC –hepatocellular carcinoma; HSC – hepatic stellate cell; IL-1 – interleukin-1, IL-6 –interleukin-6; KC – Kupffer cell; LCN2 – lipocalin 2; SNP – single nucleotide polymorphism; TGF-β – transforming growth factor beta; TNF – tumor necrosis factor; VDR – vitamin D receptor; vit D – vitamin D.

The main effects of vitamin D on processes involved in liver cancer development are summarized in Figure 1. After reviewing recently published studies, we conclude that basic studies conducted on cell lines or animals provided compelling evidence that vitamin D plays an important role in liver cancer development. On the other hand, data from clinical observational studies, although promising, are still inconclusive. Studies on the effect of vitamin D supplementation were generally focused on short-term outcomes of chronic liver diseases. There are still no reliable data on the effect of vitamin D supplementation on liver cancer occurrence or survival, and its role should be further investigated in clinical studies.
